# Influence of Structural Symmetry on Protein Dynamics

**DOI:** 10.1371/journal.pone.0050011

**Published:** 2012-11-26

**Authors:** Yasuhiro Matsunaga, Ryotaro Koike, Motonori Ota, Jeremy R. H. Tame, Akinori Kidera

**Affiliations:** 1 Special Postdoctoral Researchers Program, RIKEN Advanced Institute for Computational Science, Kobe, Japan; 2 Computational Science Research Program, RIKEN, Wako, Japan; 3 Graduate School of Information Science, Nagoya University, Nagoya, Japan; 4 Protein Design Laboratory, Yokohama City University, Yokohama, Japan; 5 Graduate School of Nanobioscience, Yokohama City University, Yokohama, Japan; King’s College London, United Kingdom

## Abstract

Structural symmetry in homooligomeric proteins has intrigued many researchers over the past several decades. However, the implication of protein symmetry is still not well understood. In this study, we performed molecular dynamics (MD) simulations of two forms of trp RNA binding attenuation protein (TRAP), the wild-type 11-mer and an engineered 12-mer, having two different levels of circular symmetry. The results of the simulations showed that the inter-subunit fluctuations in the 11-mer TRAP were significantly smaller than the fluctuations in the 12-mer TRAP while the internal fluctuations were larger in the 11-mer than in the 12-mer. These differences in thermal fluctuations were interpreted by normal mode analysis and group theory. For the 12-mer TRAP, the wave nodes of the normal modes existed at the flexible interface between the subunits, while the 11-mer TRAP had its nodes within the subunits. The principal components derived from the MD simulations showed similar mode structures. These results demonstrated that the structural symmetry was an important determinant of protein dynamics in circularly symmetric homooligomeric proteins.

## Introduction

Homooligomeric proteins have large interface areas between the subunits resulting in stable complexes [1]–[4]. Because the molecular functions of homooligomers often require their complete oligomeric forms, the overall structure of a homooligomer may help understand its molecular function [5], [6].

**Figure 1:**
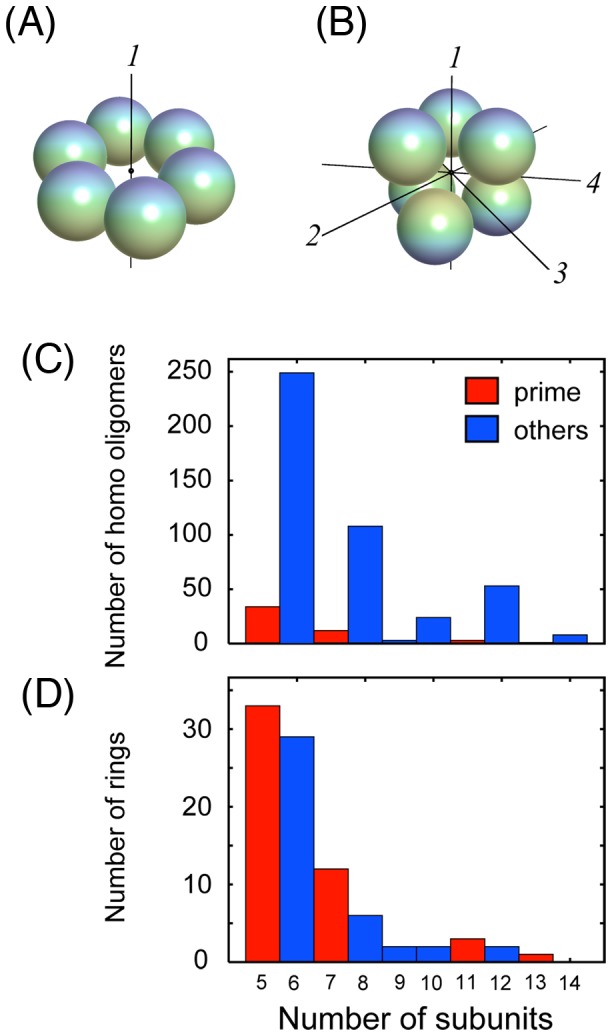
Ring and close-packed forms. (A) A schematic representation of a ring shaped oligomer. Subunits are arranged symmetrically (*C_n_* symmetry) around the rotational axis (axis *1*). Color gradation indicates the top and bottom of the subunit. (B) Schematic representation of a close-packed oligomer. The oligomer composed of *n* subunits has *n/2*-fold rotational symmetry around the axis *1*, and *2*-fold rotational symmetry around each of axes *2*–*4*. (C) The number of homooligomers (see *Materials and Methods* in detail). (D) The number of ring-shaped oligomers.

**Figure 2:**
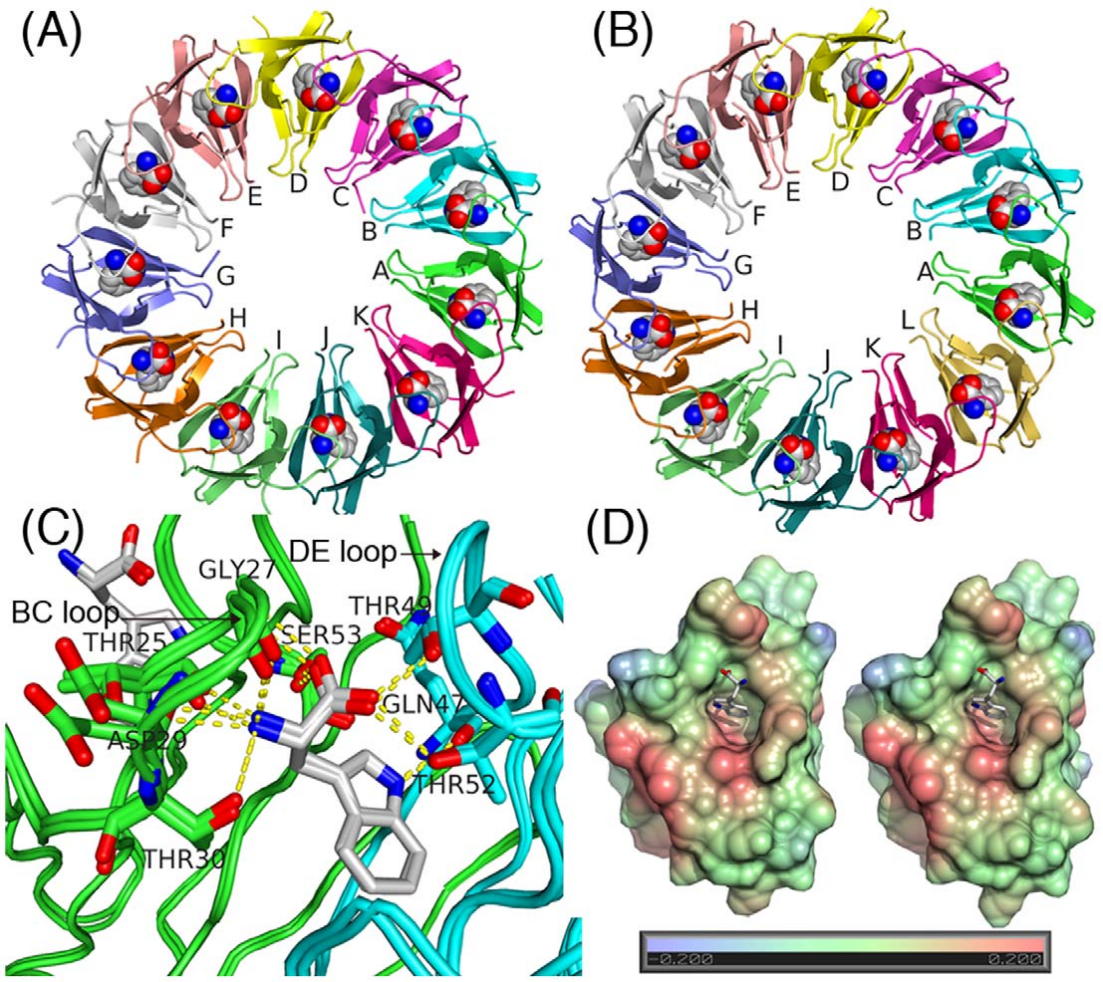
Crystal structures of the 11-mer and 12-mer TRAP. (A) Crystal structure of 11-mer TRAP (PDB code: 1C9S). Subunits and bound tryptophans are shown in ribbon and sphere, respectively. (B) Crystal structure of 12-mer TRAP (PDB code: 2EXS). (C) Superimposed structures of subunits A and B of the 11-mer and the 12-mer, shown by main-chain trace and the stick model for some side-chains. Hydrogen bonds between tryptophan and the subunit are indicated with the yellow dashed lines. (D) Hydrophobic pockets of subunit B for the 11-mer (left) and 12-mer TRAP (right). Surfaces are colored according to the hydrophobic contribution calculated by VASCo [48]. All the figures were prepared using PyMOL.

It is known that the complex structure of a homooligomer often assumes a symmetric structure [7], with the subunits arranged in either a ‘close-packed’ (or dihedral) form or a ‘ring’ form [8]. The close-packed form has *n*/2-fold rotational symmetry around one rotational axis (designated as *D_n_* where *n* is the number of subunits; axis 1 in Figure 1B) and 2-fold rotational symmetry around the other rotational axes (axes 2–4 in Figure 1B) perpendicular to the first rotational axis. Oligomers with this form contain an even number of subunits. In a statistical analysis of the Protein Data Bank (PDB) [9] (see *Materials and Methods*), we found that homooligomers composed of even numbers of subunits are dominant (Figure 1C) because of the abundance of the close-packed oligomers. In the close-packed form, the subunit interfaces are arranged in a face-to-face manner, and every structural feature or interaction is repeated twice. It was pointed out by Monod *et al.* [10] that the effect of a single mutation in complexes with the close-packed form may be much greater than in complexes without dihedral symmetry. This effect may allow such complexes to evolve more readily by the efficient generation of favorable interactions, and this prediction has been supported by recent docking-simulation studies [11]–[13].

In contrast, less attention has been paid to the minor population of ring oligomers having simple *n*-fold rotational symmetry (designated *C_n_*; Figure 1A). In our statistical analysis of the PDB, we found that such ring complexes may contain even or odd numbers of subunits, and there is no bias toward even numbers (Figure 1D). Ring-shaped oligomers have a wide variety of symmetry. Prime numbers of subunits give the “lowest” symmetry, and highly composite numbers having many divisors (such as 6 and 12) give the “highest” symmetry. A question then arises whether there is a biological or physical reason for rings to evolve with a prime number or highly composite number of subunits.

To answer this question, we studied trp RNA binding attenuation protein (TRAP) as an illustrative case. TRAP is a ring-form homooligomer for which crystal structures are available of 11-mer (prime number) and 12-mer (highly composite number) forms (Figure 2A and B). TRAP is found in various species of *Bacillus*, and plays a central role in the regulation of transcription and translation of the trp operon [14]. The monomers of TRAP form a ring-form homo 11-mer with a minor component of 12-mer depending on the solution conditions [16]–[17]. Each subunit of TRAP is composed of seven-stranded anti-parallel β-sheets and a bound tryptophan molecule. Recently, Tame *et al*. solved the crystal structure of 12-mer TRAP, which was produced artificially by joining the subunits of *B. stearothermophilus* TRAP in tandem with linkers of alanine residues [18], [19] (Figure 2B). The crystal structure of 12-mer TRAP shows exactly the same hydrogen bonding pattern and buried surface as those of the wild-type 11-mer TRAP. All-atom root mean square displacement (RMSD) between the monomer of the 11-mer and that of 12-mer was only 0.26 Å (Figure 2C and D). Despite their structural similarity, however, 12-mer TRAP is significantly less stable, as shown from the population of 12-mer in solution [15]–[17].

In this study, we tried to address the influence of the differences in symmetry on the dynamics of the oligomers. The 12-mer structure was modeled with subunits carrying no peptide linkers to stabilize the 12-mer form. We performed 100 ns fully-atomistic MD simulations with an explicit water environment for both forms of TRAPs as well as normal mode analysis using an elastic network model (ENM) [20], [21]. The normal mode analysis with group theory allows a clear description of symmetry in the thermal vibration. Based on the results of the normal mode analysis, we looked into the details of the fluctuations observed in the trajectories of the MD simulations.

## Results

### Vibrational Modes of TRAP with Perfect Rotational Symmetry: Normal Mode Analysis

**Figure 3:**
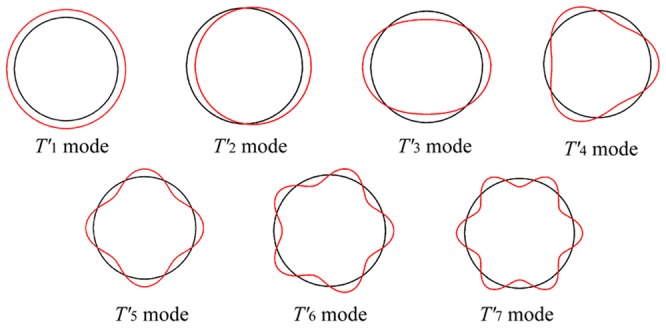
Normal modes of a ring-shaped object. Normal modes of a circularly symmetric object are viewed along the symmetry axis in the form of stationary waves on the ring. The individual mode of 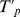 has 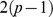 wave nodes on the ring. The red curves describe the displacements along the modes. The 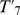 mode is found only in the 12-mer.

To characterize the vibrational fluctuations of the 11-mer and 12-mer TRAPs, we first present the group theoretical description of rotational symmetry for the two TRAPs [22]–[25]. Group theory states that a normal mode of a *C_n_* group can be viewed as a stationary wave formed by superimposing two waves propagating around the ring in opposite directions [26] (see *Materials and Methods* for details). Figure 3 shows the schematic pictures of the normal modes of the *C*_11_ and *C*_12_ groups derived from their character tables (Tables 1 and 2; these tables are given in the complex representation). For the *C_n_* group, the mode corresponding to the real irreducible representation 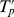 (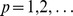) has a wave number 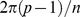 with 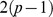 wave nodes on the ring. The nodes of a stationary wave have maximum deformations and minimum displacements while the anti-nodes have minimum deformations and maximum displacements. The complex and the real representations have the relation, 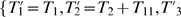 = 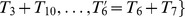 for the 11-mer and 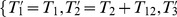 = 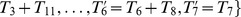 for the 12-mer. The two TRAPs share the same kinds of irreducible representations 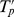 (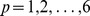) except for 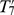 which appears only in 12-mer TRAP.

**Table 1:** Character table of 11-mer TRAP.

	*E*	*R* ^1^	*R* ^2^	*R* ^3^	*R* ^4^	*R* ^5^	*R* ^6^	*R* ^7^	*R* ^8^	*R* ^9^	*R* ^10^
*T*_1_	1	1	1	1	1	1	1	1	1	1	1
*T*_2_	1	*ω*	*ω* ^2^	*ω* ^3^	*ω* ^4^	*ω* ^5^	*ω* ^6^	*ω* ^7^	*ω* ^8^	*ω* ^9^	*ω* ^10^
*T*_3_	1	*ω* ^2^	*ω* ^4^	*ω* ^6^	*ω* ^8^	*ω* ^10^	*ω* ^12^	*ω* ^14^	*ω* ^16^	*ω* ^18^	*ω* ^20^
*T*_4_	1	*ω* ^3^	*ω* ^6^	*ω* ^9^	*ω* ^12^	*ω* ^15^	*ω* ^18^	*ω* ^21^	*ω* ^24^	*ω* ^27^	*ω* ^30^
…	…	…	…	…	…	…	…	…	…	…	…
*T*_10_	1	*ω* ^9^	*ω* ^18^	*ω* ^27^	*ω* ^36^	*ω* ^45^	*ω* ^54^	*ω* ^63^	*ω* ^72^	*ω* ^81^	*ω* ^90^
*T*_11_	1	*ω* ^10^	*ω* ^20^	*ω* ^30^	*ω* ^40^	*ω* ^50^	*ω* ^60^	*ω* ^70^	*ω* ^80^	*ω* ^90^	*ω* ^100^

Character table in the complex irreducible representation for the *C*_11_ group. *R* represents the rotation of 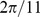 around the symmetry axis, and 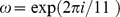. These complex irreducible representations 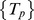 are transformed to the real, physically meaningful irreducible representations as 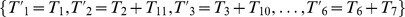.

**Table 2:** Character table of 12-mer TRAP.

	*E*	*R* ^1^	*R* ^2^	*R* ^3^	*R* ^4^	*R* ^5^	*R* ^6^	*R* ^7^	*R* ^8^	*R* ^9^	*R* ^10^	*R* ^11^
*T*_1_	1	1	1	1	1	1	1	1	1	1	1	1
*T*_2_	1	*ω*	*ω* ^2^	*ω* ^3^	*ω* ^4^	*ω* ^5^	*ω* ^6^	*ω* ^7^	*ω* ^8^	*ω* ^9^	*ω* ^10^	*ω* ^11^
*T*_3_	1	*ω* ^2^	*ω* ^4^	*ω* ^6^	*ω* ^8^	*ω* ^10^	*ω* ^12^	*ω* ^14^	*ω* ^16^	*ω* ^18^	*ω* ^20^	*ω* ^22^
*T*_4_	1	*ω* ^3^	*ω* ^6^	*ω* ^9^	*ω* ^12^	*ω* ^15^	*ω* ^18^	*ω* ^21^	*ω* ^24^	*ω* ^27^	*ω* ^30^	*ω* ^33^
…	…	…	…	…	…	…	…	…	…	…	…	…
*T*_7_	1	*ω* ^6^	*ω* ^12^	*ω* ^18^	*ω* ^24^	*ω* ^30^	*ω* ^36^	*ω* ^42^	*ω* ^48^	*ω* ^54^	*ω* ^60^	*ω* ^66^
…	…	…	…	…	…	…	…	…	…	…	…	…
*T*_11_	1	*ω* ^10^	*ω* ^20^	*ω* ^30^	*ω* ^40^	*ω* ^50^	*ω* ^60^	*ω* ^70^	*ω* ^80^	*ω* ^90^	*ω* ^100^	*ω* ^110^
*T*_12_	1	*ω* ^11^	*ω* ^22^	*ω* ^33^	*ω* ^44^	*ω* ^55^	*ω* ^66^	*ω* ^77^	*ω* ^88^	*ω* ^99^	*ω* ^110^	*ω* ^121^

Character table in the complex irreducible representation for the *C*_12_ group. *R* represents the rotation of 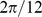 around the symmetry axis, and 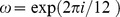. These complex irreducible representations 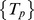 are transformed to the real, physically meaningful irreducible representations as 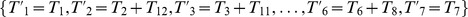.

**Figure 4:**
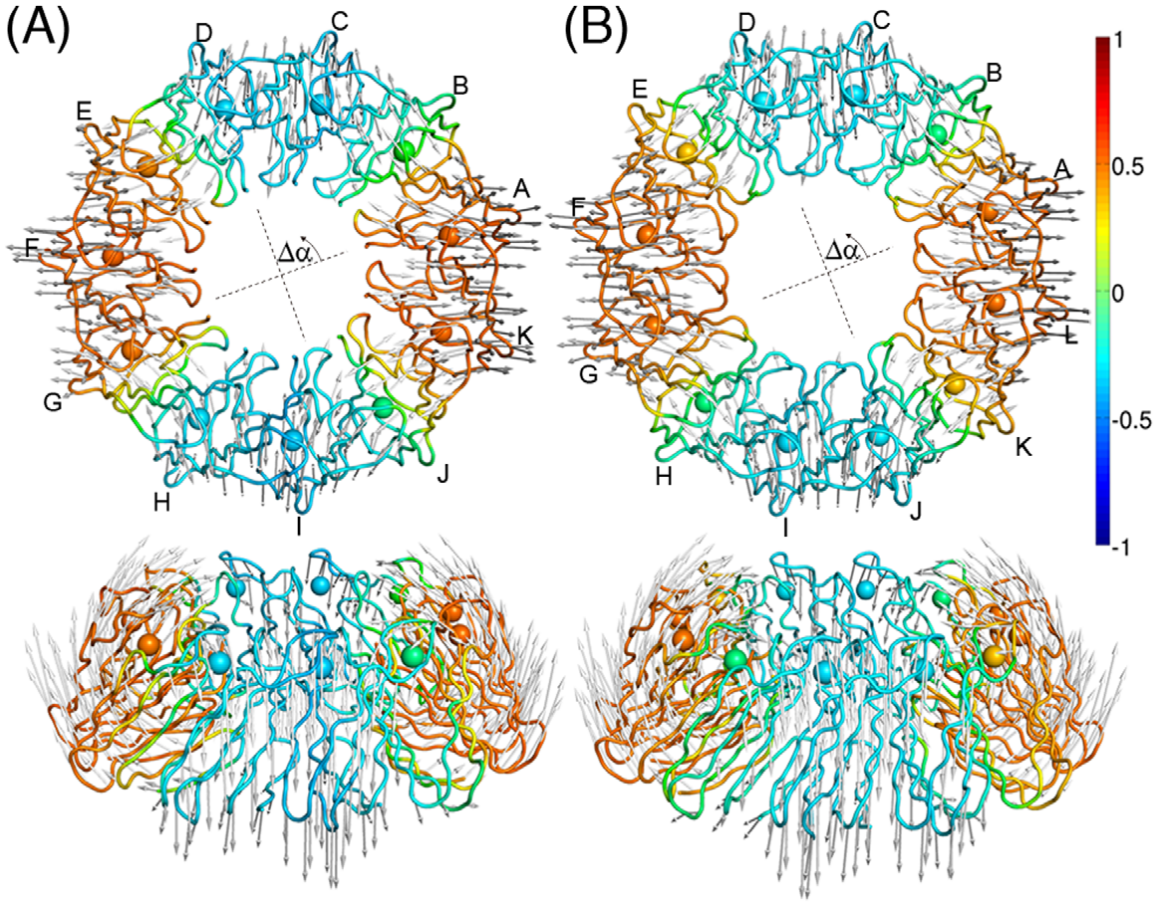
The lowest-frequency normal modes of TRAP. Top and side views of the lowest-frequency normal mode for (A) 11-mer TRAP and (B) 12-mer TRAP. The gray arrows indicate the displacements along the modes. The structures of the TRAPs are colored according to the correlation function 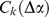 (see text and Figure 5).

Figure 4 shows the mode structures of the lowest-frequency normal modes for 11-mer and 12-mer TRAPs, derived from the normal mode analysis using the ENM with the perfectly *C_n_* symmetric systems (see *Materials and Methods*). The eigenmode structures indicate out-of-plane motions parallel to the symmetry axis (hereafter we will call it the *z*-axis). If the system could be approximated by an elastic continuum model, the motions are more and more restrained as the wave number increases. Thus, it would be expected that the lowest frequency mode belongs to the 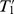 representation having no wave node, as found in the tobacco mosaic virus protein disk [26]. However, the normal mode analysis yielded the lowest-frequency mode of the two TRAPs belonging to the 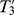 representation characterized by 4 wave nodes. In order to further investigate the differences from the elastic continuum model, we characterized the seven lowest-frequency modes. The frequency and the representation of the seven lowest-frequency modes are 0.259 (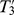), 0.259 (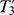), 0.341 (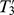), 0.341 (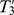), 0.462 (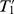), 0.553 (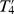) and 0.553 (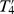) for the 11-mer, and 0.246 (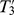), 0.246 (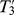), 0.313 (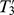), 0.313 (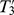), 0.452 (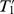), 0.535 (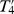) and 0.535 (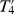) for the 12-mer (the frequency calculated by the ENM has an arbitrary unit). Here, the first and second modes, the third and fourth, and the sixth and seventh modes are degenerate pairs with shifted phases, respectively. The fifth mode looks like a uniform breathing mode which may have the lowest-frequency in the case of the elastic continuum model. The discrepancies from the elastic continuum model were also observed in the contributions of mode types to the total variance (Figure S1). In the elastic continuum model, the normal modes were classified into 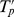, where a large value of *p* has a larger frequency, and in turn a smaller variance. However, in the case of TRAP, the normal modes classified into 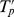 with various values of *p* had similar contributions to the total variance. This mode structure may be closely related to the shape of the normal modes on the symmetric structure of TRAP.

**Figure 5:**
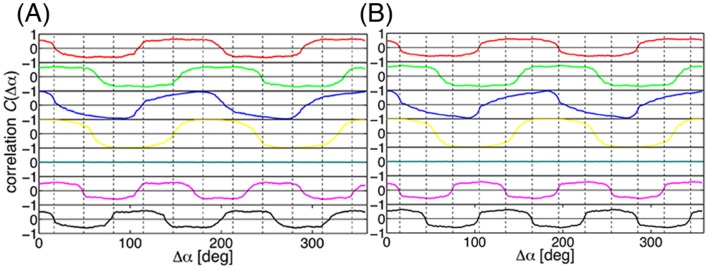
Correlations of the normal modes. Correlation function 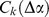 of the displacements of two atoms separated by an angle 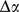 calculated for the normal modes of (A) 11-mer TRAP and (B) 12-mer TRAP. The vertical broken lines indicate the location of the subunit interfaces. The plots are for the normal modes of the 1st (red), 2nd (green), 3rd (blue), 4th (yellow), 5th (cyan), 6th (magenta), and 7th (black) from top to bottom. The pairs of normal modes, the 1st and 2nd, the 3rd and 4th, and the 6th and 7th, are 2-fold degenerate. The 5th mode is a uniform breathing mode corresponding to the 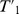 subspace.

Figure 4 also suggests positional correlation between the wave nodes and the positions of the subunit interfaces. To quantify this correlation, we defined the following correlation function after Nishikawa and Go [27] and Yu and Leitner [28], [29]:
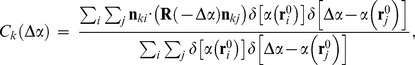
(1)


where 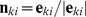 with 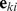 being the eigenvector of the normal mode *k* for the Cα atom *i*, 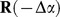 is a matrix which rotates a vector through 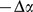 around the *z*-axis, and 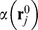 is the angular position of atom *j* around the *z*-axis with the center of mass of subunit *A* chosen as 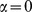. In this formula, the *δ* function has the allowance of ±4°, or 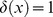 for 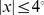 and 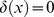 for 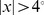. This function describes the motional correlation between the Cα atoms close to the center of mass of subunit *A* and those located at 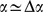 in the ring. Figure 5A and B show the values of 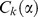 for the seven lowest-frequency normal modes of the 11-mer and the 12-mer, respectively. It was found in the 12-mer (Figure 5B) that the angles of 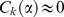; in other words, the wave nodes almost perfectly matched the position of the subunit interfaces (indicated by the broken lines) in modes 1 (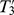), 3 (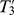), 6 (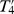), and 7 (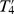). This is because the number of nodes in 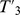 and 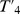, 4 and 6, respectively, are the divisors of the composite number, 12. This matching was not found in the modes 2 and 4 (the degenerated pairs of modes 1 and 2, respectively) due to the phase shift. Mode 5 is the uniform breathing 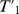 mode with no wave node. The observation that the wave nodes occur at the subunit interface may imply that the low frequency modes utilize the most weakly interacting regions, or the subunit interface, as the wave nodes. However, in the 11-mer (Figure 5A), the number of matches between the wave nodes and the subunit interfaces was about half of the number in the 12-mer. This is because the prime number of the subunits, 11, does not have an integer divisor equal to the number of wave nodes, 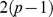, and thus some of the nodes are inevitably situated at the rigid core regions inside of the subunit.

These observations suggest that the discrepancies with the elastic continuum model appeared in the frequency and variance of the normal modes may originate from the inhomogeneity in the TRAP ring shown above, or from large deformations occurring at the subunit interfaces which are softer than the subunit cores. A normal mode having a large number of wave nodes (a large value of *p*) may move a number of subunit interfaces simultaneously to give a large deformation. However, according to the elastic continuum model, many nodes in a normal mode should increase the frequency, and decrease the variance. These two opposite effects may balance to give nearly constant variances independent of the value of *p*.

### Vibrational Modes of TRAP with Pseudo Rotational Symmetry: Molecular Dynamics Simulation

The normal mode analysis described above was based on perfectly symmetric systems. To investigate how the scenario found in the normal mode analysis works in realistic *pseudo*-symmetric systems perturbed by thermal fluctuations, we conducted two sets of 100 ns fully-atomistic MD simulations including explicit solvents for 11-mer and 12-mer TRAPs, respectively.

**Figure 6:**
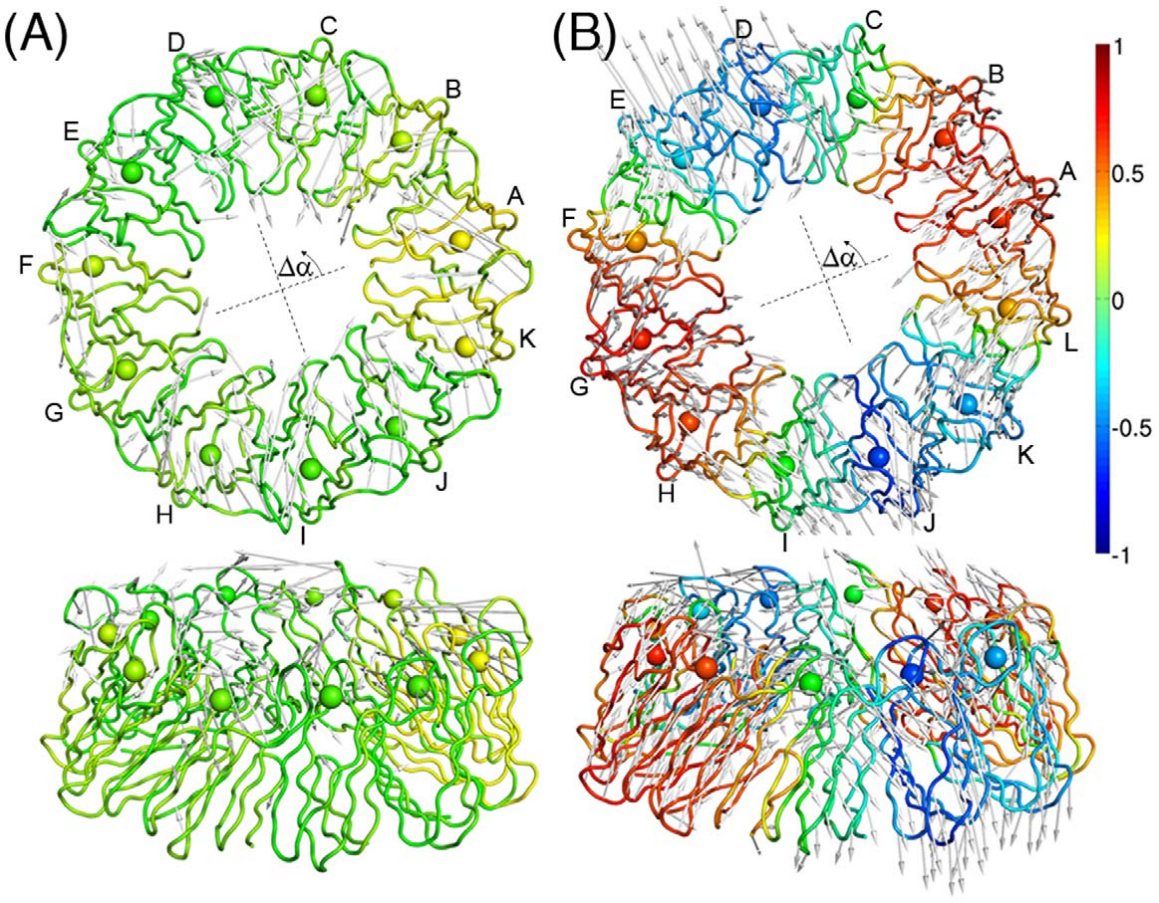
The largest-amplitude principal modes of TRAP. Top and side views of the largest-amplitude principal mode for (A) 11-mer TRAP and (B) 12-mer TRAP. The gray arrows indicate the displacements along the mode. The structures of the TRAPs are colored according to the correlation function 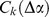 (see text and Figure 7).

To illustrate the large collective motions recorded in the MD trajectories, we carried out a principal component analysis (PCA), with the time window of 100 ns. This simulation length covers the slowest motions in the molecules, that is, 100 ns is roughly the same time-scale as one oscillation period of the first (largest-amplitude) principal mode for the 11-mer and the 12-mer. The structures of the first principal modes for the 11-mer and the 12-mer are illustrated in Figure 6A and B, respectively. For the 12-mer, we observed that the first principal mode identified by the PCA correlates with the superposition of the first and second normal modes (since the PCA is based only on the variances of data set and does not use phase information, principal modes tend to capture the superpositions of degenerated normal modes). The correlation coefficients between the first principal mode and the first and second normal modes were 0.34 and 0.60, respectively. This principal mode is also characterized by four wave nodes and the out-of-plane displacements along the *z*-axis. The simulation therefore strongly reflects the behavior observed in the normal mode analysis. For the 11-mer TRAP, however, the first mode was significantly different from the low-frequency normal modes. The first principal mode has large displacements in the BC loops (residues 25–32, facing the solvent) of several subunits, and loses large collective motions. In fact, none of the seven lowest-frequency normal modes shows significant correlation with this principal mode. The correlation coefficients between the 20 largest-amplitude principal modes and the 20 lowest-frequency normal modes are plotted in Figure S2. Although the one-to-one correspondences between the normal modes and principal modes are blurred due to the degeneracies of the normal modes, we observed correlations along the diagonal line which are weaker for the 11-mer (Figure S2A) than the 12-mer (Figure S2B).

**Figure 7:**
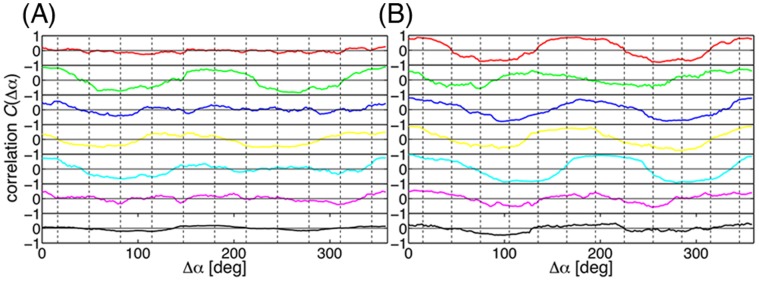
Correlations of the principal modes. Correlation function 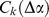 of the displacements of two atoms separated by an angle 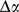 calculated for the principal modes of (A) 11-mer TRAP and (B) 12-mer TRAP. The vertical broken lines indicate the location of the subunit interfaces. The plots are for the principal modes of the 1st (red), 2nd (green), 3rd (blue), 4th (yellow), 5th (cyan), 6th (magenta), and 7th (black) from top to bottom.

In Figure 7A and B, the values of the correlation function 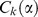, defined in Equation 1, are plotted for the seven largest-amplitude principal modes of the 11-mer and the 12-mer, respectively. As found from the normal mode analysis, the pattern of correlation in the 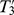 representation was also observed in both TRAPs, and tends to place the wave nodes at the subunit interfaces. However, the correlation is smaller than that found for the normal modes, particularly in the case of the 11-mer TRAP. Cooperativity of the atomic displacements around the ring can be measured by the root-mean-square (RMS) of the correlation function 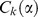, 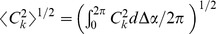. The RMS values clearly showed weaker correlation for the 11-mer (Table S1). The 11-mer had no principal modes whose RMS value exceeded 0.5, but four normal modes that did. The 12-mer had three principal modes with RMS values greater than 0.5, and six normal modes showing this level of cooperativity. The weaker cooperativity in the principal modes is due to the weakened symmetry under thermal fluctuations in the MD simulations.

**Figure 8:**
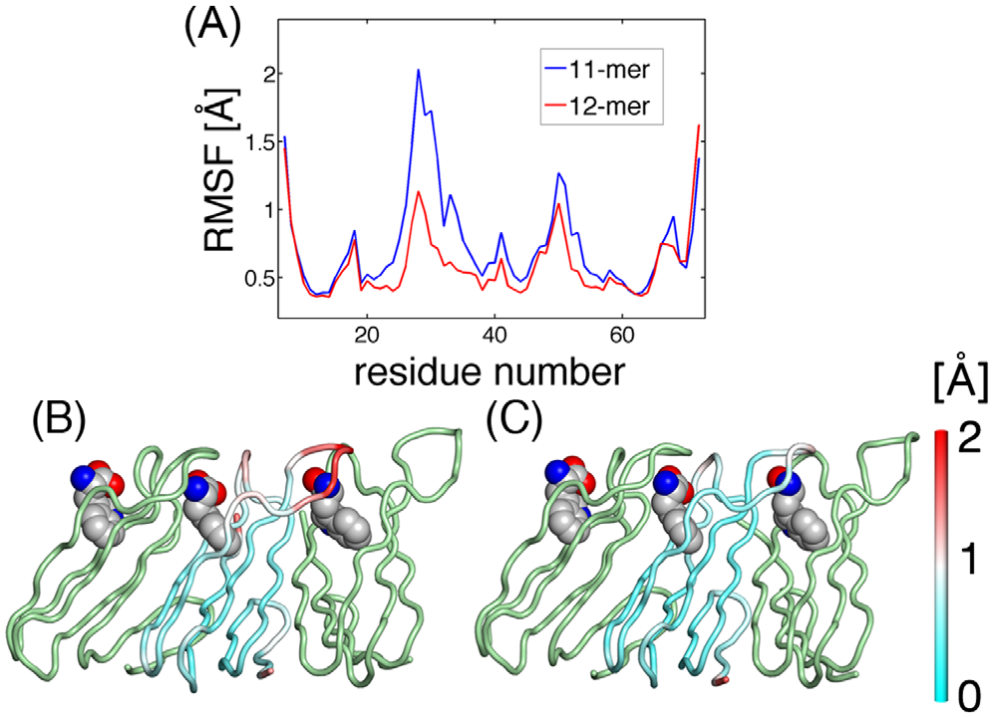
Intra-subunit fluctuations of TRAP. (A) RMS intra-subunit fluctuations of Cα atoms 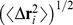 are plotted by residue for 11-mer TRAP (blue) and 12-mer TRAP (red), which are averaged over the subunits. The amplitudes of fluctuations are depicted on the structures: (B) 11-mer TRAP and (C) 12-mer TRAP. The main-chain traces are colored according to the amplitudes of the fluctuations.

The differences in the mode structures should affect the amplitude of the fluctuations of the subunits in the two TRAPs. To examine this, the RMS intra-subunit fluctuations of the Cα atoms, 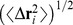 (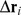 is the displacement of the Cα atom *i* from the average position), are plotted by residue in Figure 8. In this calculation, we removed the rotation and translation of a subunit by superimposing each subunit onto its average structure. As suggested by the structures of the first principal modes in Figure 6, these internal fluctuations are larger in the 11-mer TRAP than in the 12-mer. The largest differences are seen in the BC loop (residues 25–32) and the DE loop (residues 47–52). The large fluctuations in the loop regions of the 11-mer were also observed by NMR measurement [30] and a previous simulation study [31]. It was found from the MD snapshots of the 11-mer that the bound tryptophan ligand was not tightly held by its hydrogen bonds to residues on these loops. Such large loop motions were not observed in the 12-mer where the ligand molecules appeared to be firmly bound throughout the simulation. It is intriguing to find two crystal structures which are so similar, yet whose dynamics are so different. Considering the mismatch between the number of the subunits and the number of wave nodes in the 11-mer, it suggests that the fluctuations of the loops are coupled with the deformations around the wave nodes located at the subunit cores.

**Figure 9:**
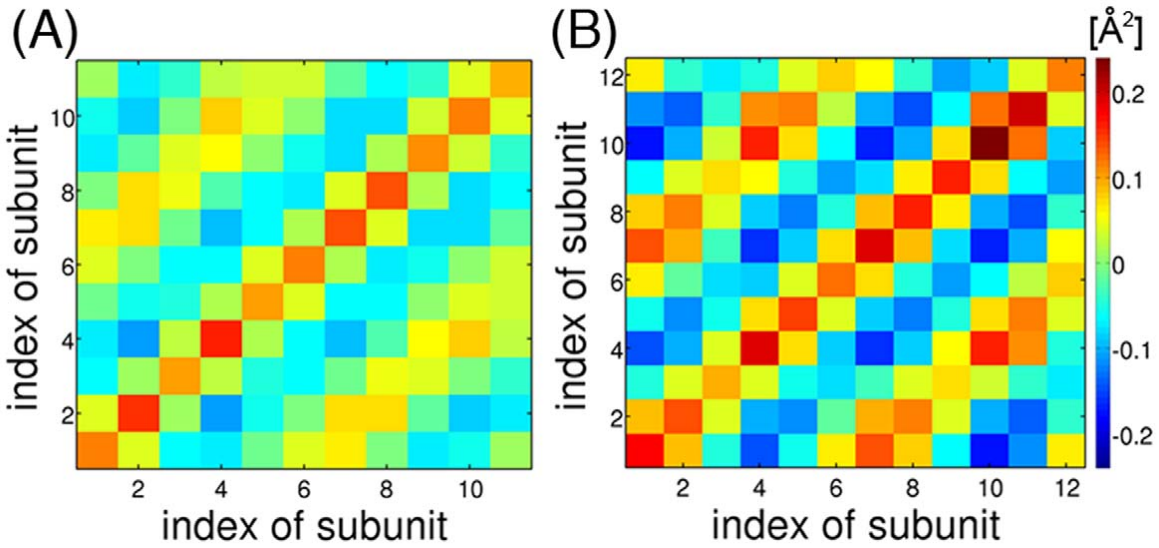
Inter-subunit correlations of TRAP. The covariance matrices of the *z*-axis component of the mass centers of the subunits are shown for (A) 11-mer TRAP and (B) 12-mer TRAP, respectively.

Figure 9 shows the covariance matrix for the *z*-components of the mass centers of the subunits,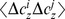, which contribute the most to the global deformations of the ring. The variances of the 12-mer (the diagonal part of Figure 9B) are larger than those of the 11-mer (Figure 9A). In both matrices, one can see positive or negative correlation between every fourth subunit, *i*, *i*+3, *i*+6, and *i*+9. The correlation between *i* and *i*+3 is negative, and between *i* and *i*+6 is positive. This pattern is characteristic in the 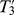 modes. In fact, essentially the same pattern was obtained using only the lowest-frequency normal modes of 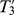. This pattern is clearer for the 12-mer than for the 11-mer since the number of subunits moving cooperatively (three) is commensurable with 12, but not with 11. Movements of the entire subunit in the *xy*-plane showed only a small difference between the two TRAPs, and their correlation pattern was found to originate from the minor 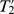 mode, not from the 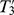 (data not shown).

**Figure 10:**
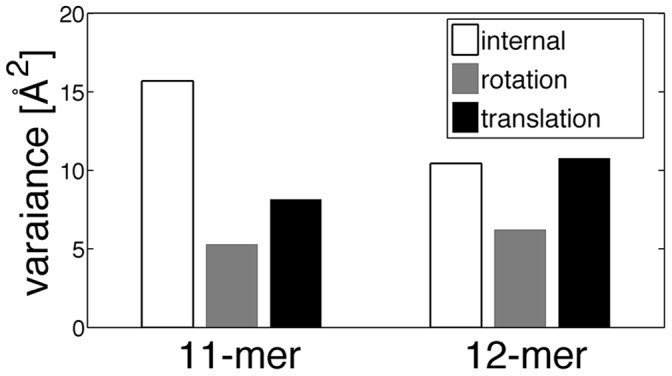
Decomposition of the subunit fluctuations into intra and external fluctuations. Intra and external (translational and rotational) subunit fluctuations in the *z*-axis are shown for the two TRAPs. The internal fluctuation was calculated after the superposition of each subunit onto its average structure, and the translational fluctuation was calculated by the variance of the center of mass of the subunit. The fluctuation of the rotation was estimated by subtracting the internal and translational contributions from the sum of the fluctuations without superimposing the subunit.

The above observations on the fluctuations were further confirmed by the decomposition of the sum of the fluctuations of the Cα atoms within a single subunit, 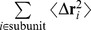, into the internal and the external (i.e., translational and rotational) contributions. The internal contribution was calculated after the superposition of each subunit onto its average structure, and the translational contribution was calculated by the variance of the center of mass of the subunit. The contribution of rotation was estimated by subtracting the internal and translational contributions from the total fluctuation. Figure 10 shows the result of the decomposition along the *z*-axis for the two TRAPs. The 12-mer has larger external (entire subunit) fluctuations than the 11-mer, while the internal (intra-subunit) fluctuation is larger in the 11-mer than in the 12-mer. The 12-mer places the wave nodes at the subunit interfaces, giving the inter-subunit motions resulting in the overall ring motions. On the other hand, the 11-mer must have wave nodes situated within the subunit core regions causing large internal deformations particularly in the loop regions.

## Discussion

The vibrational dynamics of the two TRAPs, the wild-type 11-mer and the engineered 12-mer, were investigated by focusing on their differences in rotational symmetry. First, the normal mode analysis of the perfectly symmetric TRAP system with the group theoretical approach showed that the normal modes on the ring can be viewed as a stationary wave characterized by 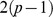 wave nodes, and that the low frequency normal modes tended to select relatively soft regions, the subunit interfaces, as the wave nodes. Because 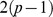 is commensurable with 12 but not with 11, the wave nodes were located at the subunit interfaces in the 12-mer, but were frequently situated at the rigid core region of the subunits in the 11-mer. This observation was utilized to study the thermally-fluctuating pseudo-symmetric systems through fully-atomistic MD simulations. In the MD snapshots, we observed similar vibrational motions as in the normal modes. In particular, large subunit interfacial deformations in the 12-mer caused larger displacements of entire subunits (external fluctuation), while in the 11-mer, wave modes located at the subunit cores caused larger intra-subunit deformations (internal fluctuation).

Generalization of these observations leads to a hypothesis that ring-form proteins of higher symmetry, with a highly composite number of subunits, undergo relatively large global deformations of the ring, and conversely that ring-form proteins with a prime number of subunits show large intra-subunit fluctuations. Each ring type may be particularly suited for different purposes where flexibility or rigidity is advantageous.

In terms of a static view of the stability of ring proteins, symmetry itself may not be a determinant of the stability. The smaller population of the 12-mer TRAP compared with the 11-mer is primarily attributed to subtle differences in the inter-subunit interactions. Antson *et al.* [32] recently found that *B. halodurans* TRAP exclusively forms a 12-mer ring. The crystal structure of the 12-mer *B. halodurans* TRAP showed the C-terminal residues with a conformation different from those of the 11-mer TRAP of *B. subtilis* or *B. stearothermophilus*, which forms different interactions with the adjacent subunit allowing an increase in the diameter of the ring [32]. However, the present study shows that symmetry significantly influences dynamics, and should be another important factor for not only stability but also biological function (for example, ligand binding) of ring proteins, especially for large ring structures like the present case of *C*_11_ and *C*_12_.

## Materials and Methods

### Dataset for Homooligomeric Proteins

We collected 1,440 complex structures of homooligomers from the PDB, determined by X-ray crystallography, and composed of at least five subunits according to PQS [33] and PISA [34]. The structures were clustered by BLASTCLUST [35] with 40% sequence identity and 80% length coverage. The structure with the highest resolution was selected as the representative from each cluster. Consequently, 495 structures were obtained in this way for analysis.

### Identification of Ring Structures

Ring structures were identified if the mass centers of subunits were located on the plane whose normal coincided with the symmetry axis of *C_n_* (Figure 1A). In practice, an oligomeric structure was judged as a candidate of having a ring structure when the third principal component calculated from the Cα coordinates was less than 2.0 Å. We obtained 106 candidates by this automatic procedure, and after visual inspection, 90 structures were identified as having ring structures. We did not include oligomers containing homodimers as the unit of the symmetry to examine effects solely due to circular symmetry.

### Elastic Network Model (ENM) and Normal Mode Analysis of TRAP

The potential energy of the ENM was defined as the sum of Hookean pairwise energy functions [20], [21], 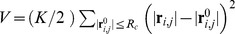, where 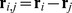 denotes the vector connecting atoms *i* and *j*, 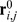 is the vector of the reference structure (see below), and *R_c_* is the cut-off distance. The strength of the potential, *K*, is an arbitrary constant assumed to be independent of the atom type. The normal modes were obtained by the diagonalization of the (mass-weighted) Hessian matrix **H** under the harmonic approximation of the potential energy, 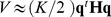, where **q** is the (mass-weighted) Cartesian coordinates of the atoms, and the superscript *t* denotes transposition. The covariance matrix **C** of the fluctuations of the Cartesian coordinates was obtained by 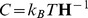, where *k_B_* is the Boltzmann constant and *T* is temperature.

In the normal mode analysis, to eliminate the influences of crystal packing and to obtain a structure with perfect rotational symmetry, we calculated a reference structure 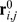 by energy minimization using the symmetry operator in the IMAGE facility of CHARMM (version c35b1) [36]. The PDB structures used in the analysis were the 11-mer wild-type TRAP from *B. stearothermophilus* (PDB code: 1C9S chain A [37]) and the engineered 12-mer TRAP (PDB code: 2EXS chain B [18]). These chains were used as the subunits of the two TRAP models. To make the chain length the same for both TRAPs, we used only the coordinates of residues 7–72, and ignored residues 1–6, 73–76, and the linker peptides in the 12-mer. In the minimization, the CHARMM 22 force field [38] with CMAP corrections [39] was used. A distance-dependent dielectric constant was applied to account for solvent screening. After the 100 steps of steepest descent minimization, the coordinates of Cα atoms in the minimized structures were used as the reference structures of the ENM. The Cα RMSD between the subunits of the 11-mer and 12-mer structures was 0.741 Å. The normal mode analysis of the ENM was performed using the symmetry basis of a Cartesian coordinate space (see below). The cut-off distance of *R_c_* = 12 Å, and *K* = 1.0 kcal mol^−1^ Å^−2^ were chosen. Changing the cut-off distance to of *R_c_* = 10 Å did not alter the result.

### Group Theory and Symmetry Coordinates

In the normal mode analysis, the symmetry of TRAP was taken into consideration based on the group theoretical approach [22]–[25] which has been used in the normal mode analysis of symmetric assemblies [26], [40], [41]. This approach represents the Hessian matrix on the basis of the symmetry coordinates. Group theory states that the symmetry coordinates are constructed with the irreducible representation of the symmetry group constituting a unique set of subspaces corresponding to the symmetry operations (rotations in the present example) [24], [25]. The irreducible representations and the corresponding character tables of the cyclic groups *C*_11_ and *C*_12_ are given in Tables 1 and 2. For the cyclic group *C_n_* with *n*-fold symmetry, the basis of the *complex* subspace 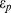 (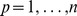) corresponding to the irreducible representation 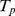 has the form [26], [41]:
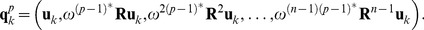
(2)


Where 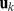 comprises an orthonormal basis for the conformation space of a single subunit 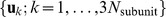 (*N*_subunit_ is the number of Cα atoms in a subunit), **R** represents the rotation of 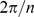 around the symmetry axis, 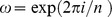, and the asterisk denotes the complex conjugate.

Since the irreducible representation 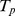 is complex, the complex subspaces 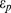 and its complex conjugate 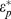 must be combined to give a physically meaningful symmetry subspace of double the dimension [26]. In the case of *C*_11_, since 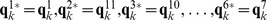, the real physically meaningful irreducible representations 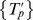 are 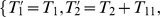 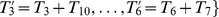. The first subspace, 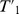, contains 3*N*_subunit_ degrees of freedom, including the global translation and rotation, while the other subspaces, 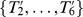, contains 6*N*_subunit_ degrees of freedom (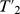 includes the translations and rotations) and doubly degenerate normal modes. For *C*_12_, they are 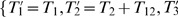 = 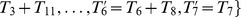. Simonson and Perahia [26] showed that a normal mode with frequency *f* in the subspace 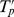 of the *C_n_* group produces a displacement of the subunit *m* of the form:
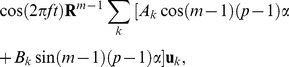
(3)


where 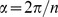, and *A_k_* and *B_k_* are constants. Equation 3 means that each normal mode of the *C_n_* group can be viewed as a stationary wave formed by superimposition of two waves propagating around the ring in opposite directions. The individual mode of 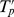 has a wave number 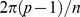 with 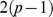 wave nodes on the ring. Schematic pictures of the 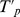 modes are illustrated in Figure 3.

### MD Simulations

The all-atom MD simulations were performed by using IBM BlueGene/L and the RIKEN Integrated Cluster of Clusters (RICC) facility. The completely symmetric structure obtained from the normal mode analysis was used as the initial structure for each TRAP. First, the structure was solvated in TIP3P water models [42] by using Solvate plugin of VMD [43] with at least 15 Å padding in each direction from the protein. We constructed a periodic box of 111×111×64 Å^3^ (73,729 atoms) for the 11-mer and 113×113×65 Å^3^ (77,958 atoms) for the 12-mer. Then, the solvent molecules and the hydrogen atoms in the protein were relaxed by a 2,000 step minimization with the backbone atoms restrained at the initial structure. After the relaxation, the system was gradually heated up from 0 K to 328 K (close to the growth temperature of *B. stearothermophilus*) in 250 ps MD simulation under the NVT ensemble. After the heating process, 100 ps simulation was performed under the NPT ensemble at 1 atm. In this stage, the backbone restraints were gradually weakened to zero. Then, the system was equilibrated in 500 ps simulation without any restraints at 328 K and 1 atm. Finally, a 100 ns production run was conducted. All the simulations were performed twice with different initial velocity conditions for each TRAP to yield two sets of 100 ns MD trajectories for each TRAP. They were qualitatively the same. All the results presented here were for one of the two. The simulations were performed using NAMD [44] with the CHARMM22 force field [38] and the CMAP corrections [39]. The particle-mesh Ewald method [45] was used to treat long-range electrostatic interactions with a direct-space cutoff of 12 Å. For temperature and pressure controls, the Langevin thermostat and barostat were used [46], [47].

## Supporting Information

Figure S1
**Contributions of the 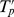 modes to the total variance.** The contributions of the normal modes to the total variance are classified according to their corresponding irreducible representations 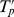. As shown in the figure, the 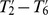 modes have similar contributions in the 11-mer and 12-mer TRAPs. The subspace spanned by the 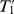 and 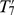 modes have a half number of degrees of freedom compared with the other modes, and thus have a half scale of the other subspaces.(TIF)

Figure S2 **Correlation between the normal modes and the principal modes.** Correlation matrices between the normal modes and the principal modes are shown for (A) 11-mer TRAP and (B) 12-mer TRAP, respectively.
(TIF)

Table S1
**RMS value of correlation function. 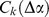.** RMS values of correlation function of the Cα atom displacements by the normal modes and the principal modes are shown for 11-mer and 12-mer TRAPs.(PDF)
