# Supplementary figures and images for: Influence of Structural Symmetry on Protein Dynamics
Source: PLoS One. 2012 Nov 26;7(11):e50011. doi: 10.1371/journal.pone.0050011 (PMC3506605; doi:10.1371/journal.pone.0050011)

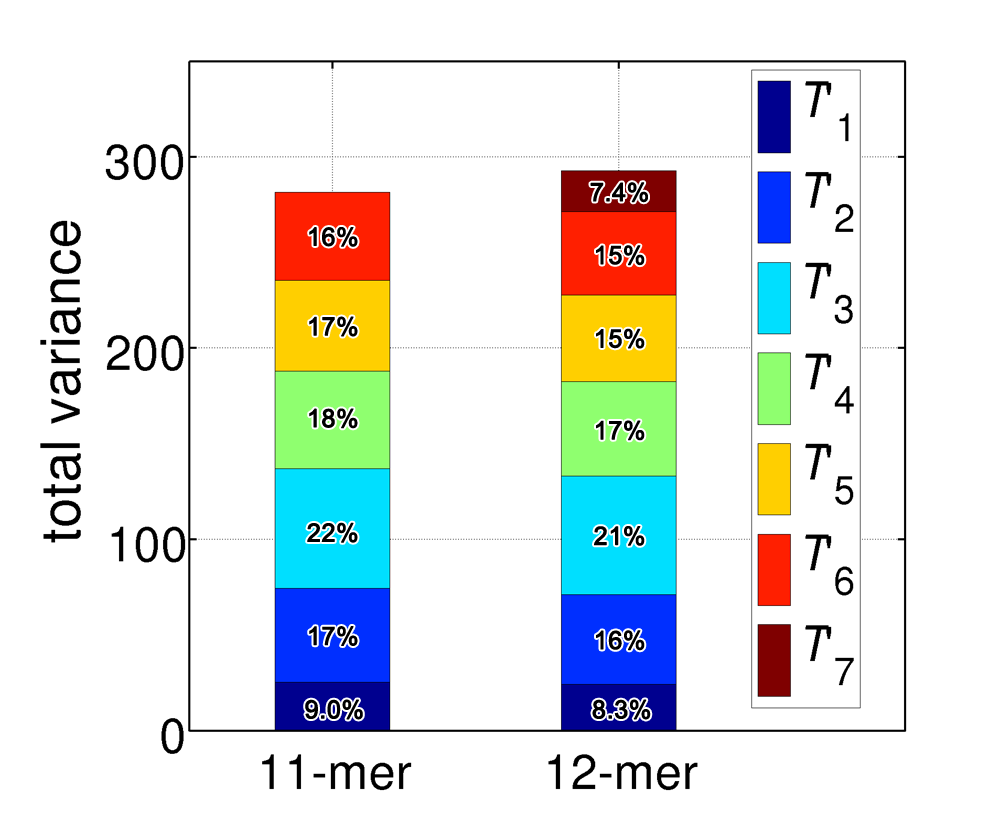

Supplement: Figure S1 — Contributions of the modes to the total variance. The contributions of the normal modes to the total variance are classified according to their corresponding irreducible representations . As shown in the figure, the modes have similar contributions in the 11-mer and 12-mer TRAPs. The subspace spanned by the and modes have a half number of degrees of freedom compared with the other modes, and thus have a half scale of the other subspaces. (TIF) [file pone.0050011.s001.tif]

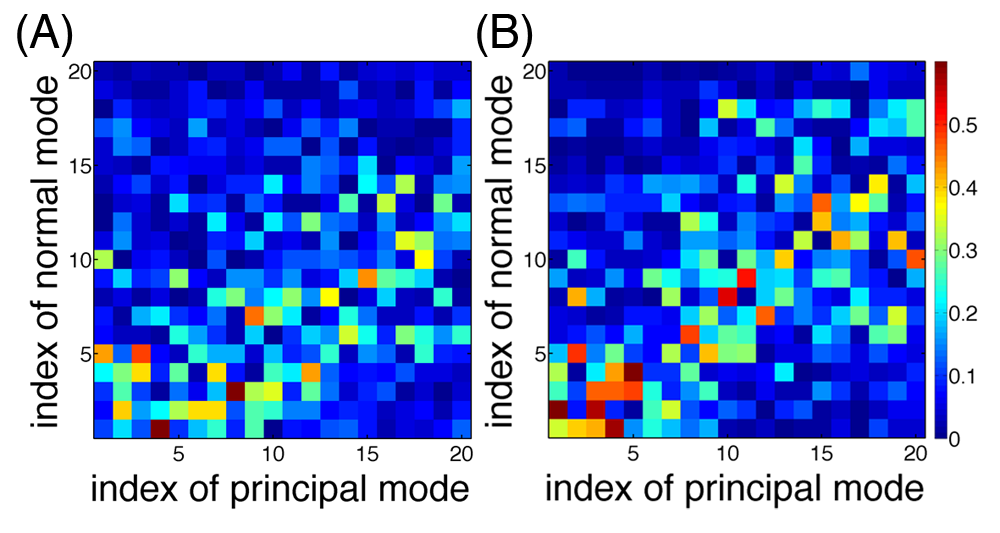

Supplement: Figure S2 — Correlation between the normal modes and the principal modes. Correlation matrices between the normal modes and the principal modes are shown for (A) 11-mer TRAP and (B) 12-mer TRAP, respectively. (TIF) [file pone.0050011.s002.tif]
