# Supplementary material for: Influence of Structural Symmetry on Protein Dynamics
Source: PLoS One. 2012 Nov 26;7(11):e50011. doi: 10.1371/journal.pone.0050011 (PMC3506605; doi:10.1371/journal.pone.0050011)
Supplement: Table S1 — RMS value of correlation function. . RMS values of correlation function of the Cα atom displacements by the normal modes and the principal modes are shown for 11-mer and 12-mer TRAPs. (PDF) [file pone.0050011.s003.pdf]

Normal modes

	1	2	3	4	5	6	7
11-mer	0.592	0.389	0.511	0.837	0.038	0.500	0.382
12-mer	0.554	0.616	0.694	0.823	0.016	0.518	0.509

Principal modes

	1	2	3	4	5	6	7
11-mer	0.347	0.358	0.297	0.437	0.086	0.363	0.455
12-mer	0.519	0.397	0.464	0.576	0.541	0.353	0.125
